# The effects of probiotic *Bacillus subtilis* on the cytotoxicity of *Clostridium perfringens* type a in Caco-2 cell culture

**DOI:** 10.1186/s12866-017-1051-1

**Published:** 2017-07-04

**Authors:** Maryam Poormontaseri, Saeid Hosseinzadeh, Seyed Shahram Shekarforoush, Tahereh Kalantari

**Affiliations:** 1 Department of Food Hygiene and Public Health, School of Veterinary Medicine, Shiraz University, P.O. Box: 71441-69155, Shiraz, Iran; 2 Diagnostic Laboratory Sciences and Technology Research Center, School of Paramedical Sciences, Shiraz University of Medical Sciences, Shiraz, Iran

**Keywords:** *Bacillus Subtilis*, *Clostridium perfringens*, Cytotoxicity, Flow cytometry, Caco-2 cell line

## Abstract

**Background:**

Some *Bacillus* strains have recently been identified for potential use as probiotics and food additives. The present study evaluated the antimicrobial effects of *Bacillus subtilis* ATCC 6633 and its metabolite on the enterotoxin and vegetative cells, spore and germinated spore of *Clostridium perfringens* type A in Caco-2 cells.

**Results:**

We used flow cytometry and MTT assays to evaluate the cytotoxicity effect of treatments. According to the results, the most cell survival was found in the 4% crude antimicrobial substance (CAS) with the vegetative form of *C. perfringens* among co-cultured groups. Furthermore, the apoptosis and necrosis in co-cultured groups were significantly decreased (*P* < 0.05).

**Conclusion:**

The present results suggested the crucial role of the current probiotic in the control of various forms of *C. perfringens* type A which was investigated for the first time. Also, the majority of treatments showed higher cell viability in flow cytometry compared to the MTT assay.

**Electronic supplementary material:**

The online version of this article (doi:10.1186/s12866-017-1051-1) contains supplementary material, which is available to authorized users.

## Background

*Clostridium perfringens* type A is the second most common cause of food poisoning in the world, and the third most common in the USA. The major virulence factor of *C. perfringens* is enterotoxin (CPE), a 35 kDa single polypeptide which causes diarrhea and abdominal cramps upon release in the host gut. CPE is a potent cytotoxic agent which acts by permeabilising enterocyte cell membranes such as Caco-2 cells by binding to claudin receptors on the apical surfaces [1].

One potential way to combat enteropathogens is through probiotics. Probiotics are microorganisms which improve humans’ and animals’ intestinal microbial balance via consumption of adequate amount of them (10^8^–10^12^ CFU/day based on type of the bacteria) [2, 3]. *Bacillus* probiotics and their metabolites are promising candidates in biotechnological applications and in the fermented foods [4]. Compared with common probiotics species such as *Lactobacillus* and *Bifidobacterium*, *Bacillus* are highly heat and chemical resistant are capable of surviving harsh gastric fluid pH due to spore forming, making them ideal as food additives for human and animal use [5]. Other beneficial effects of *B. subtilis* include production of enzymes, amino acids and antibiotic compounds such as bacteriocins and improving gut-associated lymphoid tissue (GALT) in human and animals [6, 7]. It has been previously revealed that *B. subtilis* ATCC 6633 had inhibitory effect against Gram positive and Gram negative enteropathogens via production of subtilosin A bacteriocin, in an in vitro model [7]. Furthermore, *B. subtilis* probiotics are capable of adhering the gut cells, thereby combatting enteropathogens by a competitive exclusion mechanism [7–9].

Epithelial cell monolayers such as Caco-2 and HT-29 cell lines, are well established as in vitro simulations of the gut ecosystem. These cultures are effective tools for modelling pathogen colonization and cytotoxicity in the gut, as well as studying the antibacterial effects of probiotics [10]. The present in vitro study was conducted to evaluate the antibacterial effects of *B. subtilis* ATCC 6633 on the enterotoxin, vegetative cells, spores and germinated spores of *C. perfringens* type A*.*

## Methods

### Bacterial strains and culture conditions

*Bacillus subtilis* subsp. *spizizenii* ATCC 6633 and *Clostridium perfringens* type A (NCTC 8239) was purchased from the Iranian Organizations for Science and Technology (Tehran, Iran). *B. subtilis* was aerobically cultured in TSB broth (Merck, Germany) supplemented with 1% (*w*/*v*) yeast extract (Merck, Germany) (TSBYE) for 18 h at 37 °C. *C. perfringens* was anaerobically cultured in thioglycolate broth (Merck, Germany) for 18 h at 37 °C. Both were then centrifuged (3000×g, 10 min) and washed using sterile phosphate-buffer saline (PBS, pH 7.4). A final concentration of 10^7^ CFU/mL was re-suspended in cell culture medium for further assays.

### Preparation of spore, germinated spore and CPE of *C. perfringens*

To prepare CPE, 500 mL overnight bacterial culture in thioglycolate broth (pH 7.1) was seeded into 4.5 L Duncan-Strong sporulation medium, which was in turn incubated anaerobically at 37 °C for 8 h. The prepared sporangium was then sonicated (2 min/5 min intervals) (Fungilab, Spain). The suspension was centrifuged at 10000×g for 30 min at 4 °C and the supernatant containing CPE was stored at −20 °C for SDS-polyacrylamide gel electrophoresis (SDS-PAGE) analysis. The pellet containing spores was then washed using distilled water (DW), and suspended in 20 mL DW before being stored at 4 °C. To enumerate the spores, the suspension was heated at 75 °C for 20 min, 10 fold serially diluted and subcultured onto Brain Heart Infusion (BHI) agar (Merck, Germany) and incubated anaerobically at 37 °C for 24 h [11, 12].

To vegetate spores, the spore suspension was heat activated at 75 °C for 20 min and subsequently incubated at 30 °C for 10 min [13].

### Approximate quantification of CPE and SDS-PAGE assay

To precipitate CPE, the same volume of 40% ammonium sulfate was added to the supernatant, left overnight at 4 °C, centrifuged at 10000×g/30 min/4 °C, dissolved in 0.02 M PBS and finally subjected to chromatography. Gel filtration chromatography was performed on a Sephadex G-200 gel (2 cm × 90 cm) in 0.02 M phosphate running buffer to partially purify the CPE. The flow rate was 20 mL/h and 3 mL fraction was collected [12–14]. Bradford assay was respectively employed to quantify the product. 100 μL of the preparation was initially mixed with 1 mL Bradford dye, optical density (OD) was recorded at 595 nm and the final concentration (mg/mL) was reported [15].

CPE molecular weight was then determined by SDS-PAGE based on the method described by Laemmli (1970). The CPE sample was mixed with sample buffer (62.5 mM Tris-HCl, 10% (*v*/v) glycerol, 2% (*w*/*v*) SDS, 5% (*v*/v), B-mercaptoethanol, and 0.125% (*w*/*v*) bromphenol blue, pH 6.8) and heated at 95 °C for 5 min [13].

### Preparation of antimicrobial substance

To extract the antimicrobial substance from *B. subtilis* ATCC 6633, the cell free supernatant was initially prepared by propagating the bacteria in TSBYE for 18 h at 37 °C, then centrifuged (7000×g, 20 min, 4 °C) to provide crude antimicrobial substance (CAS). The supernatant was then collected and freeze dried. Various concentrations of CAS were dissolved in RPMI medium and adjusted to pH 7 (recorded by pH meter BASIC 20, Cerison, UK). Hydrogen peroxide was removed from the CAS using catalase enzyme (Sigma-Aldrich, USA), after which the sample was filter sterilized. CAS protein concentration was determined by the Bradford method as described [15].

### Minimal inhibitory concentration (MIC) assay of CAS

8% *B. subtilis* CAS was prepared in TSB. 100 μL of the solution was then added to each well of a 96-well microtiter plate and 10 μl of the bacteria, spore and germinated spore suspensions were added to each well and incubated anaerobically at 37 °C for 24 h. Growth was determined by measuring the OD of each well at 600 nm using a micro well plate reader (BioTek, USA). MIC was confirmed at the lowest dilution of CAS without significant growth above the original inoculum (*P* < 0.05). The test was repeated in triplicate. Percentage inhibition of bacterial growth was determined using the following equation: [(OD positive control (between 0 h to 24 h) – OD sample (between 0 h to 24 h))/OD positive control (between 0 h to 24 h)] × 100 [16].

### Cell culture

Caco-2 ATCC HTB-37 was provided by the Pasteur Institute of Iran (Tehran, Iran). Cells were grown in RPMI 1640 (BioIdea, Iran) supplemented with 20% fetal bovine serum (FBS) (Gibco, USA), 1% (*v*/v) penicillin-streptomycin antibiotic (10,000 IU/mL and 10,000 μg/mL, respectively; BioIdea, Iran) and amphotericin B (50 mg/10 mL) (Sigma, USA). The Cells were incubated at 37 °C in 5% CO_2_. For subsequent assays, cell monolayers were prepared in 96-well tissue culture plates by seeding 3 × 10^4^ cells/well and incubated for 48 h to reach confluence. In Caco-2 cells, experiments were carried out after cells were differentiated (15 days post-confluence) [17].

### Cytotoxic effect of *B. subtilis* and *C. perfringens*

The cytotoxicity test was performed as previously described [18]. Briefly, the cell monolayers were first washed using PBS, and 100 μL RPMI media (not supplemented) was added to each well and incubated at 37 °C in 5% CO_2_ for 1 h. Cells were infected with 100 μL/well *B. subtilis*, CAS (4%, 8%), vegetative bacteria, spore and germinated spore of *C. perfringens* suspensions (10^7^ CFU/mL RPMI) and then incubated at 37 °C for 18 h in 5% CO_2_. Monolayers in the growth media were used as negative controls. Cellular metabolic activity assays of epithelial cells were conducted using 3-(4,5 dimethylthiazol-2-yl)-2,5-diphenyl tetrazolium bromide (MTT) kit (BioIdea, Iran).

After the incubation period, the growth medium was replaced by 100 μL RPMI 1640 (RPMI 1640 without phenol red), 10 μL 12 mM MTT solution was added to each well, plates were incubated at 37 °C for 4 h and the medium was replaced with 50 μl Dimethyl sulfoxide (DMSO). The plates were then incubated at 37 °C for 10 min and the absorbance of wells were measured at 570 nm using a microplate reader (BioTek, USA). Toxicity was calculated using the eq. [1-(OD test sample/ OD negative control)] x 100 [18].

### Cytotoxic effect of co-cultured *B. subtilis* and *C. perfringens* on Caco-2 cells

100 μL *B. subtilis* (10^7^ CFU/mL) was added to washed monolayers, which were then incubated in 5% CO_2_ at 37 °C for 1 h. 10^7^ CFU/mL of either vegetative bacterium, spore or germinated spore forms of *C. perfringens* and CPE (2 μg/mL) were then added to each well. Three wells, each of positive and negative control were also included. The microplate was then incubated in 5% CO_2_ at 37 °C for 1 h. Monolayers were then washed in sterile PBS before the MTT assay was carried out [19].

### Survival rates, early apoptosis and necrosis of treated Caco-2 cells

All treatments used in MTT assays were subjected to flow cytometry. Monolayers were incubated with *B. subtilis* probiotic (10^7^ CFU/mL) in T-12.5 flasks. 10^7^ CFU/mL each vegetative bacterium, spore and germinated spore forms of *C. perfringens* and CPE (2 μg/mL) were added to flasks containing either *B. subtilis* (10^7^ CFU/mL) or CAS (8%). Two flasks of negative (cells suspended in binding buffer 1×) and positive controls (cells added to paraformaldehyde then binding buffer) were also prepared. Cells were then trypsinized and centrifuged at 200×g, for 8 min. Supernatants were discarded and the cell suspensions were washed three times using PBS. After the final wash, supernatants were replaced by binding buffer 1× and transferred to 1.5 mL microtubes. Positive, negative and unstained controls were prepared according to the PE Annexin V Apoptosis Detection Kit I (BD Biosciences, US). For each cell experiment run, data for 100,000 events was determined by flow cytometry (BD FACSCalibur, USA) [20]. The data were then analysed using FlowJo software.

### Statistical analysis

The data were analysed using SPSS (version 16.0) software (SPSS, Inc., Chicago, IL) and subjected to one-way ANOVA test. The difference of means between the groups was also analysed using Duncan post-test. Level of statistical significance was *P* < 0.05. Flow cytometry data were also analysed by FlowJo (version 7.6) software (Flexera, US).

## Results

### Purification of CPE by SDS-PAGE

**Fig. 1:**
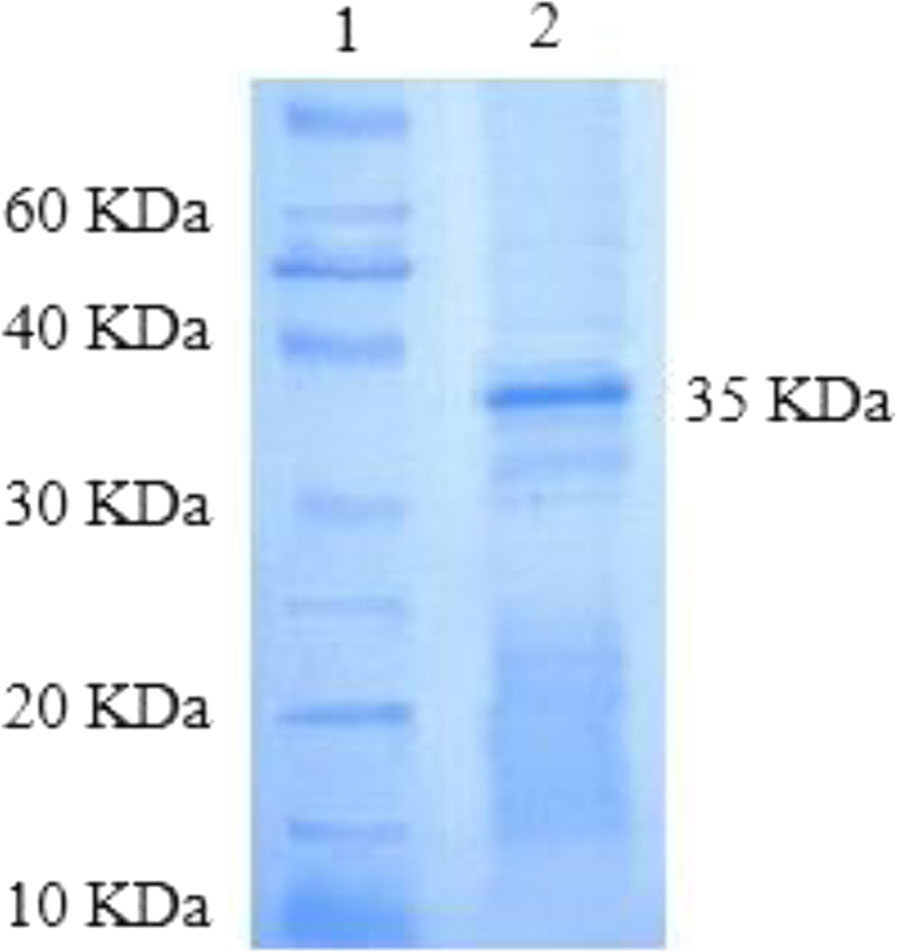
Polacrylamide gel electrophoresis of enterotoxin (50 μg) following gel filtration chromatography on Sephadex G-200. Lane 1: Protein marker (10 KDa), Lane 2: Purified CPE following chromatography

According to the protein standard curve equation of the Bradford assay (y = 91.822×-20.975, r^2^ = 0.982), a concentration of 470 μg/mL CPE was yielded by gel filtration chromatography. SDS-PAGE (10% gel) was also run to determine the corresponding molecular weight of CPE (Fig. 1).

### MIC assay of CAS

**Fig. 2:**
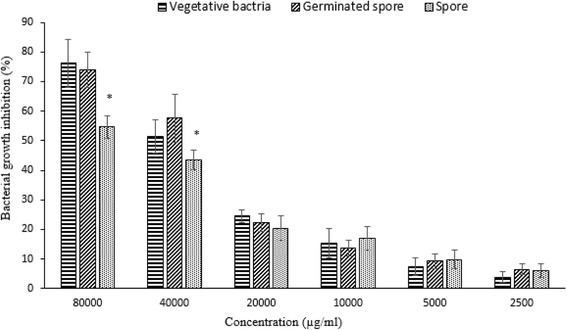
Minimal inhibitory concentration (MIC) of crude antimicrobial substance (CAS) against vegetative, spore and germinated spore of *C. perfringens* type A. *Bars* represented SD values of triplicates. Symbol (*) on the bars of each concentration representing significant differences (*P* < 0.05)

The MIC of CAS (4%, 8%) against the vegetative form of *C. perfringens* was assessed using the microdilution method. 8% CAS inhibited the growth of vegetative cells, germinated spore and spore of *C. perfringens* at the MIC of 76.12 ± 8.13%, 74.04 ± 5.99% and 54.72 ± 3.81%, respectively. At 4% CAS, MIC was 51.34 ± 5.67%, 57.63 ± 8.19 and 43.42 ± 3.39% respectively (Fig. 2). A significant difference between the vegetative bacteria, germinated spore and spore was shown in both groups.

### Cytotoxic effect of *B. subtilis* and *C. perfringens* on Caco-2 cells

The cytotoxicity of *B. subtilis*, CAS (8%, 4%), CPE (1 μg/mL) and different forms of *C. perfringens* were investigated on the Caco-2 monolayer using MTT assay. Details are given in Table 1. Caco-2 cells treated with 4% CAS were least susceptible to cytotoxic effects, while cells treated with CPE (1 μg/mL), exhibited significantly higher cytotoxicity levels (*P* < 0.05). Details are given in Table 2. Due to the high cytotoxicity observed in 8% CAS, the treatment was not continued in subsequent experiments.

**Table 1:** Different experimental groups used in this study

Experimental groups	Treatments
1	Crude antimicrobial substance (CAS) 8%
2	CAS 4%
3	*B. subtilis* ATCC 6633
4	Vegetative form of *C. perfringens*
5	Spore form of *C. perfringens*
6	Germinated spore form of *C. perfringens*
7	*C. perfringens* enterotoxin (CPE) (1 μg/mL)
8	CPE+ *B. subtilis* ATCC 6633
9	CPE+ CAS 4%
10	Vegetative form of *C. perfringens* + *B. subtilis* ATCC 6633
11	Vegetative form of *C. perfringens* + CAS 4%
12	Spore form of *C. perfringens* + *B. subtilis* ATCC 6633
13	Spore form of *C. perfringens* + CAS 4%
14	Germinated spore form of *C. perfringens* + *B. subtilis* ATCC 6633
15	Germinated spore form of *C. perfringens* + CAS 4%

**Table 2:** Percent of the cytotoxicity of *B. subtilis* strains, crude antimicrobial substance (CAS) and different forms of *C. perfringens* in Caco-2 cells

Experimental groups	Cytotoxicity (%)
1	32.09 ± 1.84^a^
2	6.1425 ± 0.82^b^
3	18.1375 ± 1.36^c^
4	46.3675 ± 1.52^d^
5	52.67 ± 1.75^e^
6	65.0425 ± 1.80^f^
7	94.9525 ± 2.91^g^

Values are mean SD of five replications. Different letters are represented the statistical difference (*P*< 0.05). Treatments are described in Table 1.

### Cytotoxic effects of co-cultured *B. subtilis* and *C. perfringens* on Caco-2 cells

The effect of *B. subtilis*, CAS, different forms of *C. perfringens* and CPE on the Caco-2 cells was assessed, both independently and in a co-culture using the MTT assay (Table 3). The highest and lowest cell viability belonged to 4% CAS and CPE (1 μg/mL) groups, respectively (*P* < 0.05). Moreover, the survival rate of cells was increased in the combined groups compared with solitary positive groups (including CPE, spore, vegetative and germinated spore) (*P* < 0.05). Among co-cultured groups, the highest and lowest cell viability was found in groups 11 and 9 respectively. Generally, co-cultured *B. subtilis* and the pathogen showed higher cell viability compared to co-cultured CAS and the pathogen.

**Table 3:** The survival rates (%) of Caco-2 cells treated with various experimental groups using MTT and flow cytometry assays

Experimental groups	Cells survival (%)	
	MTT assay	Flow cytometry assay
Negative control		97.79 ± 2.3^a^
2	95.16 ± 3.59^aA^	95.32 ± 2.77^bA^
3	86.56 ± 3.69^bA^	92.71 ± 2.77^cB^
4	56.02 ± 1.8^dA^	61.42 ± 1.21^eB^
5	51.59 ± 1.71^eA^	57.65 ± 0.83^fB^
6	37.66 ± 2^fA^	42.29 ± 0.85^gB^
7	7.05 ± 0.41^cA^	13.08 ± 0.71^dB^
8	12.74 ± 0.73^gA^	18.74 ± 2.07^hB^
9	10.94 ± 0.57^gA^	16.32 ± 1.1^jB^
10	65.55 ± 1.75^iA^	71.66 ± 1.69^kB^
11	78 ± 2.67^kA^	85.42 ± 0.87^mB^
12	59.85 ± 2.58^lA^	67.45 ± 2.05^nB^
13	72.42 ± 2.39^jA^	78.33 ± 1.89^lB^
14	44.83 ± 1.67^mA^	50.05 ± 1.74^oB^
15	51.58 ± 1.67^e^	59.40 ± 1.37^q^

Values are mean ± SD of five and three replications for MTT and flow cytometry assays, respectively. Different small and capital letters are representing the statistical difference between each column and rows, respectively (*P* < 0.05). Treatments are described in Table 1.

### Survival rates, early apoptosis and necrosis of treated Caco-2 cells

Survival rates, early apoptosis and necrosis of Caco-2 cells treated in whole groups was determined using a flow cytometry assay (Table 3). The highest cell viability belonged to the experimental group 2 (95.32 ± 2.77%) and the lowest to group 7 (13.08 ± 0.71%) (*P* < 0.05). When the probiotic strain and CAS were used in combination, the highest and the lowest survival rates of the cells changed to groups 11 (85.42 ± 0.87%) and 9 (16.32 ± 1.1%). Caco-2 cells treated with the co-cultured CAS and the pathogen revealed higher survival rates, in which, the most cell viability belonged to the experimental groups 11 (85.42 ± 0.87%), 13 (78.33 ± 1.89%) and 15 (59.40 ± 1.37%).

**Fig. 3:**
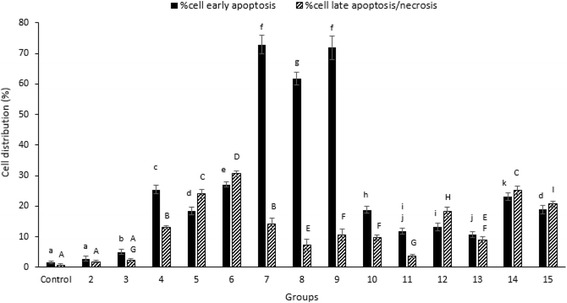
Percent of apoptotic and necrotic effects on the Caco-2 cells treated with *B. subtilis* ATCC 6633 or PY79 stains and different forms of *C. perfringens* using flow cytometry assay. Bars represented SD values of triplicates. Different small and capital letters on the bars, representing significant differences for apoptotic and necrotic cells, respectively (*P* < 0.05). Groups are described in Table 1

As shown in Fig. 3, the highest rate of apoptosis was recorded in the group 7 (79.83 ± 2.96%) and 9 (71.84 ± 3.87%) and the highest rate of necrosis belonged to the group 6 (30.6 ± 0.9%). However, the lowest apoptotic rate was found in the group number 2 (2.83 ± 0.8%). The lowest rate of necrosis was shown in the group number 2 (1.85 ± 0.52) and 3 (2.33 ± 0.56) (*P* < 0.05). In general, apoptosis rate was significantly higher or equal to the rate of necrosis in all the groups except groups 6, 12 and 14 in which, a diverse range has been observed (*P* < 0.05).

## Discussion

Our results showed *B. subtilis* bacteriocin-containing antimicrobial substance was effective against the growth of vegetative bacterium, germinated spore and spore of *C. perfringens*, with vegetative cells and germinated spores most affected in MIC assay. Due to the lower MIC required for inhibition of vegetative *C. perfringens* and the considerably high numbers of spores identified by selective staining compared with vegetative forms, this was probably the result of delayed germination of spores [21]. Ávila et al. (2013), found that the vegetative forms of *Clostridium* species exhibit higher sensitivity to ruterin and nisin than spore forms. Our result was consistent with previous reports demonstrating the inhibitory activities of nisin and partially purified bacteriocin extracted from lactic acid bacteria on the vegetative forms of *Clostridia* spp. [22, 23]. According to previous reports, lantibiotic peptides containing subtilin and subtilosin A produced by *B. subtilis* ATCC 6633, were able to inactivate numerous Gram negative and especially Gram-positive bacteria by forming voltage-dependent pores in bacterial cytoplasmic membranes [24]. Moreover, protease and pronase enzymes produced by *B. subtilis* are known to inhibit the function of CPE [25], and such proteolytic enzymes are likely present in our extract. Furthermore, it seems that a nonspecific mechanism of steric hindrance of probiotics blocks adherence of pathogens to their cell mediated receptors which is likely to be the case for CPE [7, 26]. One previous study implied that PB6, PB3 and ATCC 6633 strains of *B. subtilis* secrete anti-clostridial factors against *C. perfringens,* a causative agent of poultry necrotic enteritis, using well diffusion assay [27]. It was also found that LFB112 and 8A strains of *B. subtilis* isolated from plants and soil have inhibitory effects against *C. perfringens* via production of bacteriocin [28]. Generally, the majority of vegetative forms of *C. perfringens* strains have exhibited higher sensitivity to nisin, (a bacteriocin which is structurally and functionally similar to lantibiotic produced by *B. subtilis* ATCC 6633) than spores [29–31].

We also evaluated the cytotoxicity levels of CAS (4%, 8%), *B. subtilis*, enterotoxin and different forms of *C. perfringens.* Our findings showed that 4% CAS had the lowest cytotoxic percentage, and CPE the highest. 8% CAS also demonstrated high cytotoxicity, probably due to high levels of toxic components such as proteins in the culture medium. Our results are consistent with those of several former studies [18, 32, 33]. CPE synthesis is not only dependent on bacterial sporulation, but also can be released by spore disruption [34]. As such, the toxin can be released by spore germination. Our results revealed higher cell toxicity by the germinated spore form of C*. perfringens* compared to spore and vegetative bacteria. In agreement with earlier studies, exposing CPE (1 μg/mL) to Caco-2 and Vero cells for 15 min induced 40% and 90% of cell death, respectively. In addition, 1 μg of CPE/mL was able to induce clear morphological damage within 60 min, followed by 95% death, in Caco-2 cells [35, 36]. The ability of spore and vegetative forms of *C. perfringens* to adhere to intestinal epithelial cells was a likely avenue to causing cell damage [37]. Furthermore, our results clarified that the co-culture of probiotics or CAS and different forms of *C. perfringens*, had lower cytotoxic effects than the pathogen, using both assays. CAS which contains subtilin, subtilosin A and bacteriocin-like inhibitory substances, potentially affects the spore and vegetative forms of *C. perfringens*, suggesting the release of CPE during spore vegetation [38].

Flow cytometry was employed to further investigate the process of apoptosis and necrosis in the affected cells. Since some of the viable cells may detach from the bottom of the microplate wells during washing stage of MTT assay, the OD was frequently lower than the control group. Hence, in the majority of our experimental groups, the survival rates of Caco-2 cells were significantly higher using flow cytometry (*P* < 0.05).

Although the viability obtained from MTT and flow cytometry assays were similar, the MTT method seems to be more effective for bacterial metabolites [18, 32, 39, 40]. Due to sustained bacterial attachment, even after several washes, the OD of the remaining cells was higher following absorption of MTT stain.

Not surprisingly, the highest rate of apoptosis was demonstrated in the toxin and toxin plus CAS groups as formerly investigated [14, 41]. In general, apoptosis and necrosis in co-cultured groups were significantly lower than positive control groups (*P* < 0.05) which alludes to the beneficial effects of *B. subtilis* and its antimicrobial substance. The rate of cell necrosis and apoptosis in spore and germinated spore groups was higher than the vegetative forms which was most likely due to their higher tendency to adhere to Caco-2 cells [20, 37].

## Conclusions

This study suggested that *B. subtilis* probiotic was effective against various forms of *C. perfringens* type A in Caco-2 cell culture via antimicrobial substances. Further, we showed that the majority of treatments had higher cell viability in flow cytometry compared with the MTT assay due to higher specificity and for monitoring cell death.

However, although in vitro models are able to mimic the humans’ GI tracts, complimentary in vivo validation is also necessary.

## Additional files

Additional file 1: Chromatography of CPE. (DOC 106 kb)

Additional file 2: CPE concentration using Bradford assay. (DOC 35 kb)

Additional file 3: MIC of CAS. (DOC 40 kb)

Additional file 4: Percent of cytotoxicity (MTT assay). (DOC 32 kb)

Additional file 5: Percent of cell viability (MTT assay). (DOC 37 kb)

Additional file 6: Flow cytometry. (DOC 51 kb)

## Abbreviations

CAS: Crude antimicrobial substance
CPE: *Clostridium perfringens* enterotoxin
DW: Distilled water
MIC: Minimal inhibitory concentration
MTT: 3-(4,5 dimethylthiazol-2-yl)-2,5-diphenyl tetrazolium bromide
