# Supplementary material for: The effects of probiotic Bacillus subtilis on the cytotoxicity of Clostridium perfringens type a in Caco-2 cell culture
Source: BMC Microbiol. 2017 Jul 4;17:150. doi: 10.1186/s12866-017-1051-1 (PMC5496268; doi:10.1186/s12866-017-1051-1)
Supplement: Supplementary file 1 — Chromatography of CPE. (DOC 106 kb) [file 12866_2017_1051_MOESM1_ESM.doc]

**Additional file 1**

**Title of data: Chromatography of CPE**

| **Description of data** | |
| --- | --- |
| fraction Num. | OD |
| 1 | 0.001 |
| 2 | 0.001 |
| 3 | 0.002 |
| 4 | 0.002 |
| 5 | 0.002 |
| 6 | 0.002 |
| 7 | 0.003 |
| 8 | 0.003 |
| 9 | 0.004 |
| 10 | 0.004 |
| 11 | 0.005 |
| 12 | 0.005 |
| 13 | 0.005 |
| 14 | 0.006 |
| 15 | 0.006 |
| 16 | 0.007 |
| 17 | 0.007 |
| 18 | 0.007 |
| 19 | 0.008 |
| 20 | 0.008 |
| 21 | 0.009 |
| 22 | 0.009 |
| 23 | 0.01 |
| 24 | 0.011 |
| 25 | 0.013 |
| 26 | 0.017 |
| 27 | 0.028 |
| 28 | 0.087 |
| 29 | 0.193 |
| 30 | 0.319 |
| 31 | 0.416 |
| 32 | 0.603 |
| 33 | 0.974 |
| 34 | 1.017 |
| 35 | 1.053 |
| 36 | 1.047 |
| 37 | 0.92 |
| Cont… | |
| 38 | 0.85 |
| 39 | 0.74 |
| 40 | 0.66 |
| 41 | 0.55 |
| 42 | 0.361 |
| 43 | 0.225 |
| 44 | 0.211 |
| 45 | 0.181 |
| 46 | 0.09 |
| 47 | 0.085 |
| 48 | 0.076 |
| 49 | 0.114 |
| 50 | 0.284 |
| 51 | 0.611 |
| 52 | 0.851 |
| 53 | 0.922 |
| 54 | 1.125 |
| 55 | 1.58 |
| 56 | 1.812 |
| 57 | 1.675 |
| 58 | 1.488 |
| 59 | 0.998 |
| 60 | 0.764 |
| 61 | 0.496 |
| 62 | 0.481 |
| 63 | 0.421 |
| 64 | 0.401 |
| 65 | 0.331 |
| 66 | 0.3 |
| 67 | 0.259 |
| 68 | 0.224 |
| 69 | 0.19 |
| 70 | 0.331 |
| 71 | 0.311 |
| 72 | 0.315 |
| 73 | 0.315 |
| 74 | 0.3 |
| 75 | 0.292 |
| 76 | 0.286 |
| 77 | 0.267 |
| 78 | 0.259 |
| 79 | 0.25 |
| Cont… | |
| 80 | 0.243 |
| 81 | 0.235 |
| 82 | 0.208 |
| 83 | 0.19 |
| 84 | 0.185 |
| 85 | 0.171 |
| 86 | 0.192 |
| 87 | 0.21 |
| 88 | 0.222 |
| 89 | 0.21 |
| 90 | 0.204 |
| 91 | 0.196 |
| 92 | 0.173 |
| 93 | 0.15 |
| 94 | 0.12 |
| 95 | 0.11 |
| 96 | 0.1 |
| 97 | 0.08 |
| 98 | 0.07 |
| 99 | 0.12 |
| 100 | 0.11 |
| 101 | 0.108 |
| 102 | 0.1 |
| 103 | 0.07 |
| 104 | 0.05 |
| 105 | 0.041 |
| 106 | 0.009 |
| 107 | 0.009 |
| 108 | 0.006 |
| 109 | 0.004 |
| 110 | 0.004 |
