# Supplementary material for: The effects of probiotic Bacillus subtilis on the cytotoxicity of Clostridium perfringens type a in Caco-2 cell culture
Source: BMC Microbiol. 2017 Jul 4;17:150. doi: 10.1186/s12866-017-1051-1 (PMC5496268; doi:10.1186/s12866-017-1051-1)
Supplement: Supplementary file 2 — CPE concentration using Bradford assay. (DOC 35 kb) [file 12866_2017_1051_MOESM2_ESM.doc]

**Additional file 2**

**Title of data: CPE concentration using Bradford assay ‎**

| **Description of data** | |
| --- | --- |
| OD | concentration |
| 0.237 | 0 |
| 0.243 | 1 |
| 0.247 | 3 |
| 0.26 | 4 |
| 0.294 | 5 |
| 0.298 | 6 |
| 0.318 | 8 |
| 0.329 | 9 |
| 0.368 | 13 |
| 0.387 | 15 |
| 0.403 | 16 |
