# Supplementary material for: The effects of probiotic Bacillus subtilis on the cytotoxicity of Clostridium perfringens type a in Caco-2 cell culture
Source: BMC Microbiol. 2017 Jul 4;17:150. doi: 10.1186/s12866-017-1051-1 (PMC5496268; doi:10.1186/s12866-017-1051-1)
Supplement: Supplementary file 3 — MIC of CAS. (DOC 40 kb) [file 12866_2017_1051_MOESM3_ESM.doc]

**Additional file 3**

**Title of data: MIC of CAS**

| **Description of data** | | | | | | |
| --- | --- | --- | --- | --- | --- | --- |
| ***C. perfringens* forms** | **CAS concentration** | | | | | |
| Vegetative bacteria | **80000** | **40000** | **20000** | **10000** | **5000** | **2500** |
|  | 73.64 | 44.86 | 26.87 | 11.38 | 7.86 | 5.88 |
|  | 69.54 | 53.78 | 22.94 | 20.82 | 10.14 | 3.25 |
|  | 85.24 | 55.38 | 23.58 | 13.57 | 4.1 | 2.43 |
| Mean values | 76.14 | 51.34 | 24.4633333 | 15.25667 | 7.366667 | 3.853333 |
| Germinated spore | 80.64 | 66.65 | 19.57 | 11.57 | 11.28 | 4.68 |
|  | 68.92 | 50.64 | 25.64 | 16.61 | 6.48 | 6.47 |
|  | 72.57 | 55.62 | 21.58 | 12.55 | 9.91 | 8.28 |
| Mean values | 74.04333 | 57.63666667 | 22.2633333 | 13.57667 | 9.223333 | 6.476667 |
| Spore | 58.45 | 46.71 | 30.57 | 16.77 | 12.58 | 8.61 |
|  | 50.84 | 39.92 | 37.19 | 22.47 | 10.35 | 4.12 |
|  | 54.87 | 43.63 | 29.44 | 14.82 | 6.48 | 5.14 |
| Mean values | 54.72 | 43.42 | 32.4 | 18.02 | 9.803333 | 5.956667 |
