# Supplementary material for: The effects of probiotic Bacillus subtilis on the cytotoxicity of Clostridium perfringens type a in Caco-2 cell culture
Source: BMC Microbiol. 2017 Jul 4;17:150. doi: 10.1186/s12866-017-1051-1 (PMC5496268; doi:10.1186/s12866-017-1051-1)
Supplement: Supplementary file 4 — Percent of cytotoxicity (MTT assay). (DOC 32 kb) [file 12866_2017_1051_MOESM4_ESM.doc]

**Additional file 4**

**Title of data: Percent of cytotoxicity (MTT assay)**

| **Description of data** | | | | | | | |
| --- | --- | --- | --- | --- | --- | --- | --- |
| **Different forms of bacteria** | | | | | | | |
| Cytotoxicity | B. sub6633 | CAS 4% | CAS 8% | C. per | spore | germinated spore | toxin |
|  | 16.22 | 5.21 | 28.7 | 46.23 | 54.14 | 65.41 | 95.6 |
|  | 18.12 | 6.45 | 27.12 | 46.35 | 52.92 | 64.83 | 94.87 |
|  | 19 | 6.91 | 35.7 | 45.64 | 51.74 | 64.76 | 92.83 |
|  | 19.21 | 6 | 36.84 | 47.25 | 51.88 | 65.17 | 96.51 |
| Mean values | 18.1375 | 6.1425 | 32.09 | 46.3675 | 52.67 | 65.0425 | 94.9525 |
