# Supplementary material for: The effects of probiotic Bacillus subtilis on the cytotoxicity of Clostridium perfringens type a in Caco-2 cell culture
Source: BMC Microbiol. 2017 Jul 4;17:150. doi: 10.1186/s12866-017-1051-1 (PMC5496268; doi:10.1186/s12866-017-1051-1)
Supplement: Supplementary file 5 — Percent of cell viability (MTT assay). (DOC 37 kb) [file 12866_2017_1051_MOESM5_ESM.doc]

**Additional file 5**

**Title of data: Percent of cell viability (MTT assay)**

| **Description of data** | | | | | | | | | | | | | | |
| --- | --- | --- | --- | --- | --- | --- | --- | --- | --- | --- | --- | --- | --- | --- |
| **Experimental groups** | | | | | | | | | | | | | | |
| Cell viability | CAS 4% | B.sub6633 | C.per | spore | germinated spore | toxin | toxin+6633 | toxin+4% | c.per+6633 | C.per+4% | spore+6633 | spore+4% | Germinated spore+6633 | Germinated spore+4% |
|  | 95.24 | 86.23 | 55.54 | 51.44 | 36.46 | 6.6 | 13.52 | 9.91 | 64.41 | 77.57 | 60.88 | 71.84 | 43.12 | 52.44 |
|  | 96.12 | 86.76 | 56.35 | 52.44 | 38.72 | 7.13 | 12.7 | 11.83 | 65.79 | 77.64 | 60.67 | 72.71 | 44.73 | 51.69 |
|  | 94.18 | 87.44 | 55.22 | 51.76 | 37.29 | 7.57 | 12.84 | 10.66 | 65.44 | 78.57 | 58.91 | 72.55 | 46.95 | 51.33 |
|  | 95.1 | 85.83 | 56.98 | 50.74 | 38.19 | 6.89 | 11.89 | 11.35 | 66.56 | 78.24 | 58.93 | 72.58 | 44.54 | 50.86 |
| Mean values | 95.16 | 86.565 | 56.0225 | 51.595 | 37.665 | 7.0475 | 12.7375 | 10.9375 | 65.55 | 78.005 | 59.8475 | 72.42 | 44.835 | 51.58 |
