# Supplementary material for: The effects of probiotic Bacillus subtilis on the cytotoxicity of Clostridium perfringens type a in Caco-2 cell culture
Source: BMC Microbiol. 2017 Jul 4;17:150. doi: 10.1186/s12866-017-1051-1 (PMC5496268; doi:10.1186/s12866-017-1051-1)
Supplement: Supplementary file 6 — Flow cytometry. (DOC 51 kb) [file 12866_2017_1051_MOESM6_ESM.doc]

**Additional file 6**

Title of data: Flow cytometry

| **Description of data** | | | | | | | | | | | | | | | |
| --- | --- | --- | --- | --- | --- | --- | --- | --- | --- | --- | --- | --- | --- | --- | --- |
| **Experimental groups** | | | | | | | | | | | | | | | |
| Cell viability | **control** | **CAS 4%** | **B.sub 6633** | **C.per** | **spore** | **germinated spore** | **toxin+B.sub 6633** | **toxin+4%** | **C.per+B.sub 66** | **C.per+4%** | **spore+B.sub 6633** | **spore+4%** | **germ spore+B.sub66** | **germ spore+4%** | **toxin** |
|  | 98.32 | 96.21 | 93.21 | 60.42 | 57.12 | 42.21 | 19.52 | 14.81 | 71.29 | 86.22 | 68.25 | 77.35 | 51.6 | 59.78 | 13.35 |
|  | 98.68 | 94.94 | 91.83 | 61.64 | 58.61 | 43.18 | 18.86 | 17.53 | 73.51 | 85.55 | 66.02 | 78.31 | 50.44 | 59.49 | 12.75 |
|  | 96.27 | 94.81 | 93.11 | 62.21 | 57.21 | 41.48 | 17.84 | 16.61 | 70.18 | 84.49 | 68.08 | 79.33 | 48.11 | 58.94 | 13.14 |
| mean values | 97.75667 | 95.32 | 92.71667 | 61.42333 | 57.64667 | 42.29 | 18.74 | 16.31667 | 71.66 | 85.42 | 67.45 | 78.33 | 50.05 | 59.40333333 | 13.08 |
| Necrosis | 0.71 | 2.47 | 2.88 | 13.54 | 23.42 | 30.01 | 6.42 | 9.82 | 9.58 | 4.1 | 17.11 | 9.32 | 24.57 | 20.58 | 14.52 |
|  | 0.43 | 2.04 | 1.49 | 12.54 | 25.55 | 31.63 | 7.21 | 10.3 | 10.61 | 4.24 | 17.54 | 9.64 | 21.73 | 21.73 | 15.28 |
|  | 0.59 | 1.05 | 2.61 | 13.28 | 22.86 | 30.12 | 8.23 | 11.81 | 8.67 | 2.55 | 19.98 | 7.86 | 22.88 | 19.63 | 12.34 |
| mean values | 0.576667 | 1.853333 | 2.326667 | 13.12 | 23.94333 | 30.58666667 | 7.286666667 | 10.64333 | 9.62 | 3.63 | 18.21 | 8.94 | 23.06 | 20.64666667 | 14.04667 |
| Apoptosis | 2.21 | 2.68 | 5.94 | 23.92 | 18.21 | 27.51 | 62.85 | 74.97 | 18.86 | 9.87 | 13.92 | 10.88 | 25.87 | 20.45 | 72.91 |
|  | 0.83 | 3.64 | 4.18 | 25.62 | 19.63 | 26.14 | 61.55 | 70.71 | 19.73 | 10.89 | 13.54 | 10.64 | 23.76 | 19.41 | 73.82 |
|  | 1.84 | 2.18 | 4.73 | 26.81 | 17.3 | 27.55 | 60.72 | 72.85 | 17.54 | 12.1 | 15.54 | 12.58 | 26.45 | 17.82 | 71.89 |
| Mean values | 1.626667 | 2.833333 | 4.95 | 25.45 | 18.38 | 27.06666667 | 61.70666667 | 72.84333 | 18.71 | 10.95333 | 14.33333333 | 11.36667 | 25.36 | 19.22666667 | 72.87333 |
